# Internal Subdivisions of the Marmoset Claustrum Complex: Identification by Myeloarchitectural Features and High Field Strength Imaging

**DOI:** 10.3389/fnana.2019.00096

**Published:** 2019-11-27

**Authors:** Xiuxian Pham, David K. Wright, Nafiseh Atapour, Jonathan M.-H. Chan, Kirsty J. Watkins, Katrina H. Worthy, Marcello Rosa, Amy Reichelt, David H. Reser

**Affiliations:** ^1^Alfred Health, Melbourne, VIC, Australia; ^2^Department of Neuroscience, Central Clinical School, Monash University, Melbourne, VIC, Australia; ^3^The Florey Institute of Neuroscience and Mental Health, Parkville, VIC, Australia; ^4^Department of Physiology, Biomedicine Discovery Institute, Monash University, Clayton, VIC, Australia; ^5^Australian Research Council Centre of Excellence for Integrative Brain Function, Monash University Node, Clayton, VIC, Australia; ^6^Robarts Research Institute, Western University, London, ON, Canada; ^7^Graduate Entry Medicine Program, Monash Rural Health, Churchill, VIC, Australia

**Keywords:** claustrum, marmoset, non-human primate, myelin, myeloarchitectonics, forebrain and brainstem afferent pathways

## Abstract

There has been a surge of interest in the structure and function of the mammalian claustrum in recent years. However, most anatomical and physiological studies treat the claustrum as a relatively homogenous structure. Relatively little attention has been directed toward possible compartmentalization of the claustrum complex into anatomical subdivisions, and how this compartmentalization is reflected in claustrum connections with other brain structures. In this study, we examined the cyto- and myelo-architecture of the claustrum of the common marmoset (*Callithrix jacchus*), to determine whether the claustrum contains internal anatomical structures or compartments, which could facilitate studies focused on understanding its role in brain function. NeuN, Nissl, calbindin, parvalbumin, and myelin-stained sections from eight adult marmosets were studied using light microscopy and serial reconstruction to identify potential internal compartments. Ultra high resolution (9.4T) post-mortem magnetic resonance imaging was employed to identify tractographic differences between identified claustrum subcompartments by diffusion-weighted tractography. Our results indicate that the classically defined marmoset claustrum includes at least two major subdivisions, which correspond to the dorsal endopiriform and insular claustrum nuclei, as described in other species, and that the dorsal endopiriform nucleus (DEnD) contains architecturally distinct compartments. Furthermore, the dorsal subdivision of the DEnD is tractographically distinguishable from the insular claustrum with respect to cortical connections.

## Introduction

The claustrum is a bilateral subcortical structure located between the insular cortex and basal nuclei of most mammalian species, and characterized by widespread connectivity to the neocortex. There has been growing attention and interest surrounding its structure and function over the past decade, spurred by the hypothesis that this nucleus plays a role in critical sensory processes underlying consciousness (Crick and Koch, 2005). Since the publication of that landmark article, there has been a steady increase in the understanding of structural-functional relationships, especially those associated with claustrum-neocortex connections. A number of alternative hypotheses regarding claustrum function have been proposed, including the salience-attention hypothesis (Mathur et al., 2009; Remedios et al., 2014; Smythies et al., 2014; Goll et al., 2015), and the network hypothesis (Patru and Reser, 2015; Reser et al., 2017). The majority of anatomical and hodological work on the claustrum has been performed in rodents, which has produced several of the leading hypotheses surrounding claustrum function (Mathur et al., 2009; Smith et al., 2017; Watson et al., 2017; White et al., 2018). However, several as yet unexplained differences in claustrum-cortex connectivity have been reported between rodents and primates (Smith and Alloway, 2010; Reser et al., 2017; Smith et al., 2017).

Anatomical descriptions of the claustrum complex have reported that it is a relatively homogenous structure, consisting of only a few well-defined cell types (Braak and Braak, 1982; Rahman and Baizer, 2007; Kim et al., 2016). A few biochemical and genetic markers of claustrum boundaries have also been reported across species, including: the carboxypeptidase inhibitor latexin [Arimatsu et al., 2009 (cat); Watakabe et al., 2014 (macaque and rodent); Orman, 2015 (rodent); Orman et al., 2017 (bat)]; netrin-G2 [Miyashita et al., 2005 (macaque); Pirone et al., 2012 (human)]; and the gamma-2 subunit of guanine binding protein [GNG-2; Mathur et al., 2009 (rat); Pirone et al., 2012; Watakabe et al., 2014], and the nuclear receptor subtype of the transcription factor *nurr-1* (Watakabe et al., 2014). However, the expression of these markers is also somewhat variable across species. Moreover, Watakabe et al. (2014) and Watakabe (2017) have suggested that there is internal compartmentalization of expression of claustrum markers in non-human primates, which may reflect internal anatomical subdivisions of the complex. Two major subdivisions—the claustrum and the endopiriform nucleus, are recognized based on cytoarchitecture, with additional subdivision of the endopiriform nucleus into dorsal and ventral components (Hardman, 2012; Paxinos et al., 2012). Yet, despite variations of expression of marker genes within the marmoset claustrum, no marker clearly conformed to the compartmental boundaries described in the stereotaxic atlases (Watakabe et al., 2014). It is also notable that the large and well-developed claustrum complex in the short-tailed fruit bat (*C. perspicillata*) exhibits differential immunoreactivity for latexin between the claustrum and endopiriform nucleus. Thus, additional insight into internal claustrum organization is likely to be obtained from observations of species other than rodent or primate models (Orman et al., 2017).

Crucially, compartmentalization of the claustrum has been reported based upon developmental patterns of gene expression in mice (Watson and Puelles, 2017; Binks et al., 2019) and in nonhuman primates (Binks et al., 2019). Cells of the insular claustrum and dorsal endopiriform nucleus (DEnD) express the Nr4a2 prenatally in mice, indicating they are derived from the lateral pallium cell population. In contrast, cells of the ventral endopiriform nucleus do not express Nr4a2, and originate in the ventral pallium (Watson and Puelles, 2017). Recent work by the same group has shown that this developmental pattern is largely conserved in primates, despite a more complex adult topology of the claustrum complex (Binks et al., 2019). This complexity has led to uncertainty in the identification of claustrum complex compartments in primates in the past, although there has been a recent consensus regarding the broad divisions of the complex (dorsal endopiriform and insular claustrum) in marmosets and macaques (Smith et al., 2019). It is possible that different regions of the claustrum have different functions, similar to other large subcortical complexes such as the amygdala and the thalamus. A firm grasp on the internal compartmentalization of the claustrum is desirable, as a necessary anatomical guide for designing experiments aimed at testing the various hypotheses on its function.

Here, we describe the myeloarchitectural, cytoarchitectural, and chemoarchitectonic features of the claustrum complex in the common marmoset (*Callithrix jacchus*), a small New World monkey species. In addition, we describe the tractographic profiles of two constituent nuclei of the claustrum complex (insular claustrum—CLA; dorsal division of the DEnD) based on ultra-high resolution whole-brain post-mortem imaging. The marmoset is an increasingly popular model for neuroanatomical, hodological, and connectomic studies, as it retains important characteristic features of the cortices of larger primates, including humans (Solomon and Rosa, 2014; Tokuno et al., 2015; Majka et al., 2016). Harmonization of nomenclature for comparative studies of the claustrum complex was a key objective of the recent review by Smith et al. (2019), and the present report attempts to follow as closely as possible the conventions and consensus views of claustrum structure that were outlined in this article.

## Materials and Methods

Eight post-mortem marmoset brains were obtained from adult marmosets involved in other experiments in our laboratory (Atapour et al., 2017, 2018), for which approval was obtained from the Monash University Animal Ethics Committee. Case details are presented in Table 1. Six of the animals were euthanized with sodium pentobarbital (100 mg/kg, ip) and transcardially perfused with 0.1 M PBS followed by 4% paraformaldehyde (PFA). Brains were extracted, post-fixed in 4% PFA overnight, and cryoprotected in serially increased sucrose concentrations (10–30% in 4% PFA). Cryoprotected brains were blocked and coronally sectioned at 40 μm thickness using a cryostat.

**Table 1:** Case details.

Animal ID	Weight (g)	Age (years)	Sex
CJ167	390	2	F
CJ170	326	2	M
CJ173	340	2	M
CJ189	305	2	F
F1741	295 g	1.5	M
F1882	300 g (est.)	3	F
CJ194	360	2	M
CJ197	395	2	F

Two cases (CJ194 and CJ197) were processed using a similar protocol to that described above, except that ice-cold artificial cerebrospinal fluid was substituted for the 0.1 M PBS and 4% PFA in the perfusion solution, and the unfixed brain was extracted and immersed whole in 4% PFA for 2 days, after which post-mortem MRI scanning was performed using a 9.4T/20 cm Bruker MRI. Case CJ197 was subsequently immersed in 4% PFA with 0.1% Gadovist gadolinium contrast solution (Bayer Australia, Pymble, NSW, Australia) for a separate imaging study. For imaging, each brain was immersed in proton-free oil (Krytox, Sigma–Aldrich) and multi-gradient echo images (T_2_*-weighted) were acquired using a 40 mm volume resonator (Bruker) and the following imaging parameters: repetition time (TR) = 120 ms; echo times (TE) = 8, 16,…, 48 ms; field of view (FOV) = 32.50 × 25.6 × 25.6 mm^3^; matrix size = 650 × 512 × 512; and resolution = 50 × 50 × 50 μm^3^. The total imaging time was approximately 9 h. After acquisition, the individual echo images were averaged and the mean echo T_2_*-weighted image used for subsequent analyses. The mounting process was similar to the method of Luciano et al. (2016).

Diffusion-weighted imaging (DWI) was also performed for case CJ197 using a 6-shot echo planar imaging based sequence with the following imaging parameters: TR = 700 ms; TE = 33 ms; FOV = 32 × 26.25 × 24 mm^3^; matrix = 256 × 210 × 192; resolution = 125 × 125 × 125 μm^3^; diffusion duration = 5 ms; diffusion separation = 15 ms; *b*-value = 5,000 s/mm^2^; diffusion directions = 81; non-diffusion images = 1; total imaging time was approximately 18 h. The fiber orientation distribution (FOD) was estimated using constrained spherical deconvolution and whole-brain fiber tracking performed using MRtrix3 software^1^ (Tournier et al., 2004).

The claustrum and endopiriform nuclei were outlined on the mean T_2_*-weighted image using ITK-SNAP^2^ and the resulting segmentations registered to the DWI using Advanced Normalization Tools (ANTs^3^). Additionally, the NIH Marmoset Brain Atlas connectivity-based parcellation was also registered to the DWI and the number of streamlines connecting each cortical region to both the claustrum and dorsal division of the DEnD segmentations were counted.

The same streamline analyses could not be performed on the brain from case CJ194, due to cortical tissue damage resulting from an implanted electrode array used for* in vivo* electrophysiological recordings. These recordings resulted in perforated regions of tissue within visual areas V1 and MT that would be likely to distort estimates of streamline projections to caudal cortex, so claustro-cortical connections could not be quantified.

After all imaging procedures were completed, the brains were rinsed in 4% PFA for 24 h, then cryoprotected and sectioned for histology in the same manner as the other cases.

Adjacent sections were stained for myelin using the Gallyas silver method (Gallyas, 1979). In five cases (CJ167, CJ170, CJ173, CJ189, CJ194) neuronal nuclei were stained by immunohistochemistry using anti-neuronal nuclear protein (anti-NeuN) primary antibody (1:800, MAB377, clone A60, Merck Millipore, Burlington, MA, USA) at 4°C for 42–46 h. This was followed by incubation in secondary antibody (1:200, PK-6102, Vectastain Mouse IgG kit, Vector Laboratories, Burlingame, CA, USA) for 30 min and enhancement with the streptavidin-horseradish peroxidase DAB method (DAB peroxidase Substrate kit SK4100, Vector Laboratories, Burlingame, CA, USA). Immunoreactivity in marmoset brain tissue has been previously reported for this commercial antibody (Leuner et al., 2007; Sawamoto et al., 2011; Atapour et al., 2018). Negative control sections processed without the primary antibody yielded no NeuN positive nuclei. Complete immunohistochemical methods for NeuN staining are described in Atapour et al. (2018).

Case F1741 was immunostained for calbindin and case F1882 was stained for parvalbumin using previously described procedures (Bourne et al., 2007). Briefly, tissue sections were washed three times in 0.1 M PBS, and then blocked in a solution of 0.1 M PBS; 0.3% Triton X-100; and 10% normal horse serum for 1 h at room temperature. After blocking, the primary antibody (Swant Swiss mouse monoclonal anti-calbindin D-28k, code no. 300; 1:8,000 dilution; or Swant Swiss mouse monoclonal anti parvalbumin, code 235; 1:8,000 dilution) was added and sections were incubated at 4°C for 40–48 h. At the conclusion of the primary antibody immersion, sections were washed three times in 0.1 M PBS and incubated in 0.1 M biotinylated anti-mouse secondary antibody (Vectastain ABC Elite kit PK6102, Vector Laboratories, Burlingame, CA, USA) at room temperature for 30 min. Immunoreactivity was visualized using the ABC reagent system enhanced with DAB (DAB kit SK-4100, Vector Laboratories, Burlingame, CA, USA). After the DAB reaction, sections were mounted on glass slides, dried for approximately 48 h, and coverslipped with DPX mounting medium for slide scanning and light microscopy.

In three cases (F1741, F1882, CJ197) neuronal cell bodies were stained for Nissl substance using the cresyl violet technique, then dried and coverslipped for scanning. Sections from case CJ197 were cut and mounted parasagittally but were otherwise processed as described above.

Histological and immunostained sections were scanned at 20× using an Aperio Scanscope AT Turbo color scanner (Monash Histology Platform, Monash University, Clayton, VIC, Australia). Acquired images were batch converted from the native format to the JPEG-2000 format using custom software.

For semi-quantitative analyses of calbindin- and parvalbumin-positive claustrum cells, scanned images were overlayed with internal claustrum boundaries as determined from the adjacent myelin sections in Illustrator CS6. Immunopositive cell bodies were counted using the “Document Info: Objects” function in Illustrator, and cell density was computed by dividing each count by the area of the respective subdivision as computed using the AreaLength.js Illustrator script^4^.

Images of histological sections and individual MRI sections were captured as either .TIF, .JP2 or .PNG files using a Zeiss Axioplan2 light microscope with a Zeiss ICc5 camera or on a Leica DMS1000 upright microscope with MC170-HD camera and 2× achromat objective, then cropped and adjusted for brightness and contrast in Adobe Photoshop (v. CS6, Adobe Systems, Mountain View, CA, USA). MRI sections were filtered once using the “sharpen” function in Photoshop to allow adequate comparison to histological sections. Images were aligned, labeled, and exported to their final format in Adobe Illustrator (v. CS6, Adobe Systems, Mountain View, CA, USA). All image manipulations (e.g., brightness/contrast adjustments) were applied to the entire tissue section in a non-destructive manner. In some cases, minor histological artifacts such as tears, or folded portions of sections, were retained in the final image. Original image files are available on request.

Three-dimensional serial reconstructions of stacked images were generated in FIJI (Schindelin et al., 2012). Static images were captured for illustration from rotating .gif movie files. The original .gif format files are available on request.

## Results

**Figure 1:**
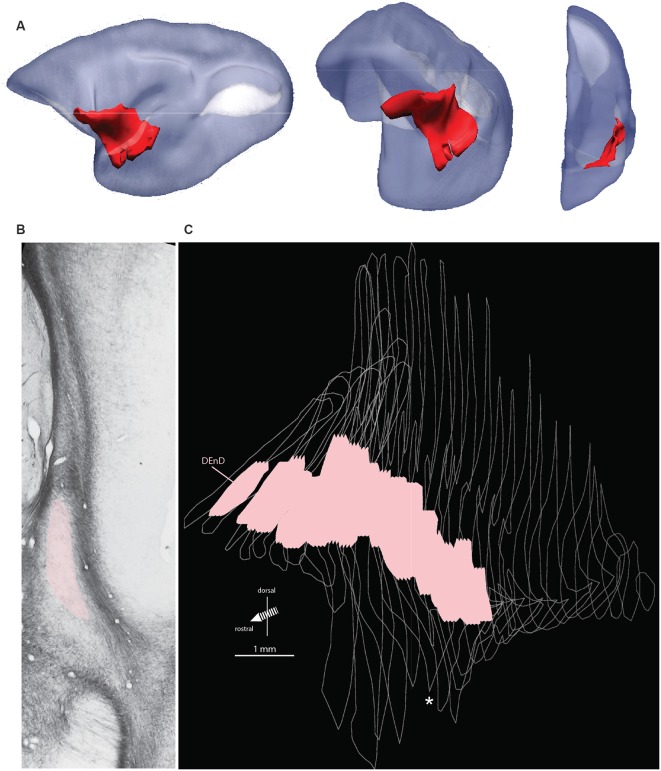
Orientation, location, and internal boundary of the marmoset claustrum. Panel **(A)** shows the lateral, oblique, and front views (left to right, respectively) of the marmoset claustrum complex in red, reconstructed from serial histological sections. The relative lissencephaly of the marmoset brain is evident, with the two major cortical sulci, the lateral sulcus (dark) and calcarine sulcus (white) visible in the lateral view. Note the significant change in the orientation of the claustrum complex axes from rostral to caudal, which can make comparison of e.g., medial or ventral regions difficult across the length of the structure. Panel **(B)** shows a representative myelin stained section from case CJ167. Adjacent structures, including the lateral nucleus of the amygdala, insular cortex, and putamen, are indicated, and the myelin-sparse dorsal subdivision of the dorsal endopiriform nucleus (DEnD) is highlighted in pink. Panel **(C)** shows the serial reconstruction of the claustrum complex from wireframe outlines of myelin sections in case CJ167 (performed using ImageJ software). The pink region within each section indicates the boundary of the DEnD region as identified in the myelin series. Jagged edges on the dorsal and ventral surface of the pink regions indicate interpolation artifacts from the slice registration. Inter-section interval is approximately 200 μm per slice. The wireframe reconstruction is oriented at approximately the same frontal oblique angle as the middle section of **(A)**. The white asterisk indicates the position of the section shown in **(B)**. Note that an enlarged and parcellated figure containing the tissue section in **(B)** appears as a preliminary data figure in Smith et al. (2019; Figure 9).

The marmoset claustrum extends approximately 7 mm along the rostral-caudal axis of the brain and extends approximately 9 mm along the dorsal-ventral axis at its broadest point. The claustrum complex is easily resolved from surrounding forebrain structures along most of its span, with the exception of the rostral most 200–500 μm, and at its lateral-most extent, where it is closely adjacent to, and possibly contiguous with, the cortical concavity at the fundus of the lateral sulcus. Topologically, the sheet-like claustrum complex changes orientation along its length (Figure 1A). In coronal sections, its long axis was oriented mediolaterally at the rostral edge, changing smoothly to a dorsal-ventral orientation at approximately the rostral extent of the amygdala in the coronal plane of section. This warping of the claustrum structure can result in confusion of anatomical reference, such that the lateral terminus of the rostral claustrum complex is adjacent to the dorsal edge of the mid-claustrum. Similarly, the medial portion of the rostral claustrum complex corresponds to the ventral edge of the middle and caudal regions of the complex. These transitions are evident in the 3-dimensional reconstruction shown in Figure 1A, and in the claustrum outline silhouettes in Figures 3A–G.

**Figure 2:**
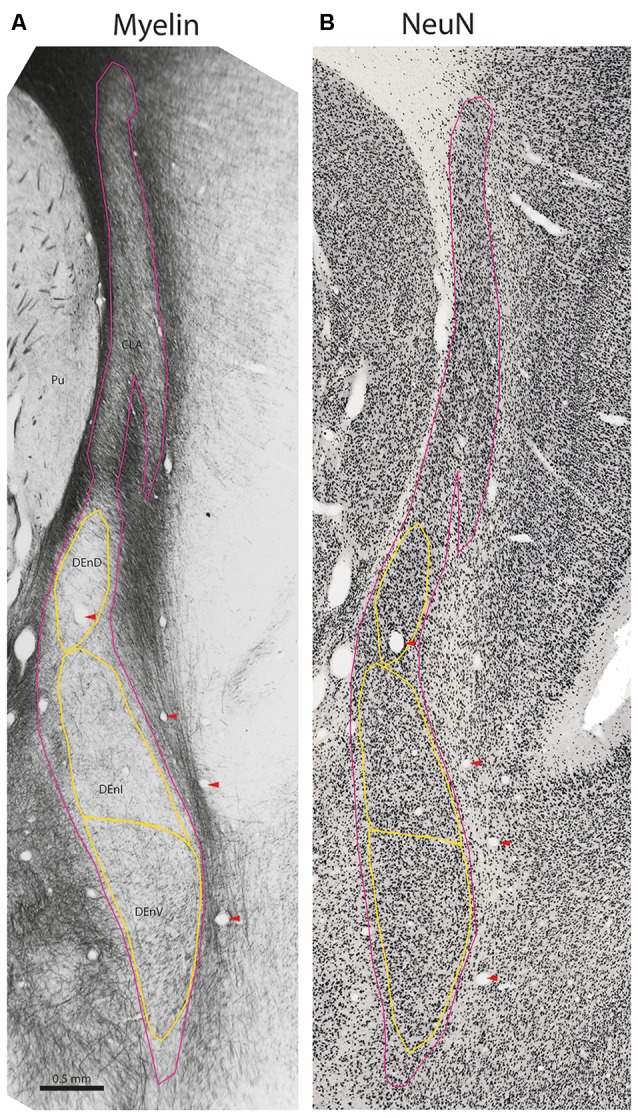
Internal subdivisions of the claustrum complex in the marmoset. Adjacent coronal sections stained for myelin (Gallyas, 1979) and the neuronal marker NeuN (Atapour et al., 2018), respectively. The observed internal boundaries of the claustrum complex, including the proposed subdivisions of the DEnD, are outlined in yellow **(A)**. Note that the sparse myelin in the DEnD region corresponds to an area of relatively high-density NeuN staining **(B)**, but the DEnI and DEnV boundaries are not well resolved in NeuN, which was consistent across the sections examined in this study. Red arrowheads indicate blood vessels used for registration of adjacent stained sections. Case CJ167, approximate A-P +10.3 (Paxinos et al., 2012). CLA, insular claustrum; DEnD, DEnI, DEnV, dorsal, intermediate and ventral subdivisions of the dorsal endopiriform, respectively; Pu, putamen.

**Figure 3:**
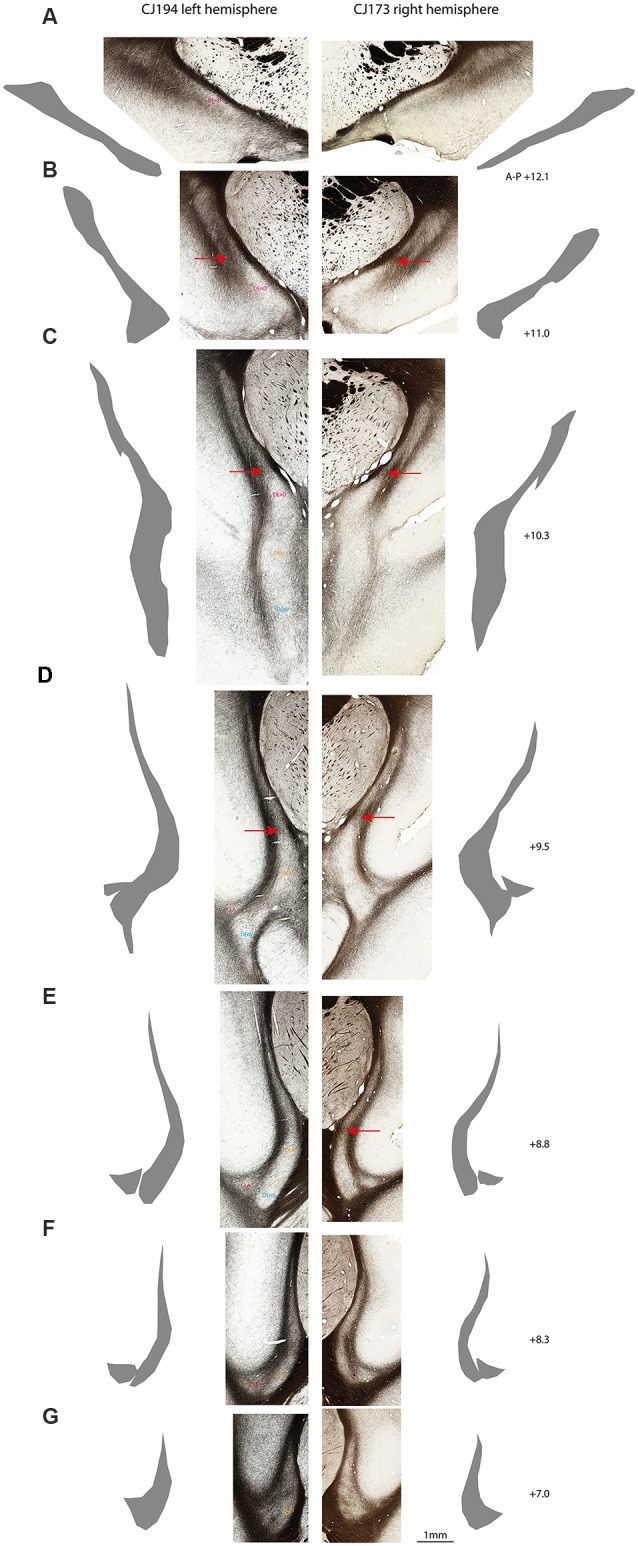
Myeloarchitectonic parcellation of the claustrum complex in two marmoset hemispheres. Rostral **(A)** to caudal **(G)** coronal sections from the left cerebral hemisphere of case CJ194 (left column) and the right hemisphere of case CJ173 (right column). Adjacent silhouettes show hand-demarcated claustrum complex masks for each section. (**B–E)** Red arrows indicate the transverse myelin band which appears to connect the external and extreme capsules, and which is concordant with the dorsal boundary of the DEnD, where present. **(F)** CLA and DEn subdivisions are indicated by the text in the left column, with the right column left unmarked for unobstructed display. Approximate A-P stereotaxic positions based on the Paxinos et al. (2012) atlas are indicated to the right of each pair of sections.

### Myeloarchitecture

The claustrum is lightly myelinated throughout its rostral-caudal axis, allowing differentiation between the claustrum complex and its adjacent white matter tracts, the external and extreme capsules (Figures 1B, 2A). Within the claustrum complex, we identified four subdivisions—a discontinuous region corresponding to the classical "insular claustrum" (CLA), and dorsal, intermediate, and ventral subdivisions of the (DEnD, DEnI, and DEnV, respectively). Each subdivision was associated with clear changes in the density and orientation of myelinated axons within and traversing the claustrum complex.

Although the insular claustrum is much less densely myelinated than the adjacent external and extreme capsules, it represents the only subdivision with consistent intrinsic myelination (i.e., short, well-stained axons which do not exhibit consistent directionality). The insular claustrum is comprised of a dorsal, sickle-shaped region adjacent to the insular cortex, which follows the curvature of the extreme capsule into the fundus of the lateral sulcus (Figures 1B, 2A). At the ventral boundary of the insula, corresponding roughly to the border of the proisocortical auditory area in the Paxinos atlas (Paxinos et al., 2012), the insular claustrum spreads into a triangular extension of the claustrum complex. In the coronal plane of section, this region is generally separated from the remainder of the claustrum complex by a myelinated tract approximately 0.1–0.2 mm in diameter. This myelin band typically extends 0.2–0.5 mm along the rostral-caudal axis of the claustrum, resulting in an apparent discontinuity in the claustrum complex. The density of myelin within this triangular discontinuity is generally consistent with the myelination observed in the thin upper segment of the insular claustrum (Figures 3E,F).

Within the DEnD, there is a small oval region of sparse myelin that appears early in the rostral sections, terminating at approximately two-thirds of the length of the claustrum caudally. This region closely corresponds to an area of dense NeuN staining and was identified as the DEnD (Figures 1B, 2). We performed a 3D reconstruction of this region in relation to the remainder of the claustrum complex from serial coronal sections of the right hemisphere (Figure 1C).

Inferior to the DEnD is an area of light myelin staining with myelin fibers appearing to run almost parallel along the longitudinal axis of the nucleus. There is an area of slightly increased myelin staining at the ventral-most aspect of the nucleus, with fibers that appear to run transversely along the medial-lateral axis of the claustrum complex. We identified the former area as the intermediate endopiriform nucleus and the latter as the ventral endopiriform nucleus. The DEnI and DEnV subdivisions were best visualized in the myelin series. In NeuN stains, the borders between the DEnI and DEnV were not well resolved. It should also be noted that the boundary enclosing the full extent of the claustrum complex does vary somewhat, depending upon whether it is identified by myelination, as in Figure 3, or on cell-body staining as in the NeuN stained section shown in Figure 2B.

We consistently observed a transverse band of myelin that appears to connect the external and extreme capsules. The ventral edge of this myelin tract is adjacent to the dorsal extent of the DEnD, although it is not clear whether this band actually forms the dorsal boundary of the DEnD. This tract is readily observed in all of the cases examined to date, although it is most apparent in the coronal plane of section (Figures 3B–E). The consistency of myeloarchitecture patterns between claustrum complex subdivisions is illustrated in Figure 3, which shows representative coronal sections from two cases, one from the right hemisphere (CJ173) and one from the left (CJ194), taken from approximately the same levels along the anterior-posterior axis of the claustrum. The silhouettes of each claustrum were hand-rendered in Adobe Illustrator, and were used to create the 3-d reconstructions of the claustrum complex, as well as the masks used for tractographic analysis in DWI cases.

### Quantitative Analysis of Calbindin and Parvalbumin Distribution

**Figure 4:**
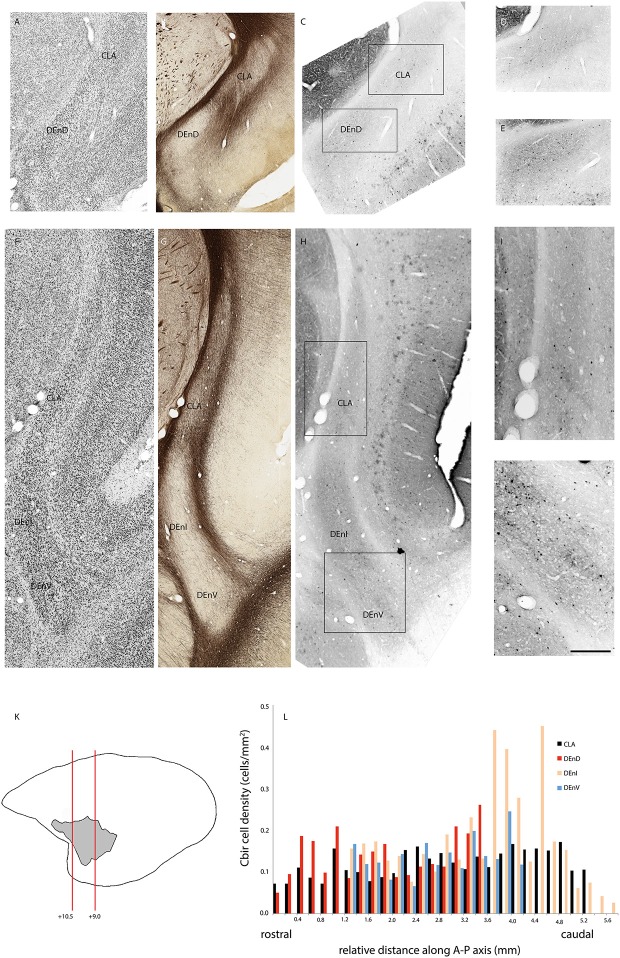
Adjacent rostral- **(A–E)** and mid- **(F–J)** coronal sections of the claustrum complex showing Nissl **(A,F)**; myelin **(B,G)**; and calbindin immunoreactive **(C–E,H–J)** staining in the CLA and DEnD subdivisions at each level. Rectangles in **(C)** and **(H)** indicate the boundaries of the enlarged images in **(D–E)** and **(I–J)**. Note that the DEnD is not visible in (**F–H)**. Red lines in **(K)** indicate the location and approximate A-P coordinates for each set of sections in **(A–J).** Bar graph in **(L)** shows the density of calbindin immunopositive cells in each subdivision of the claustrum complex for every section in this case (F1741) in which claustrum components were present. X-axis distances were computed with reference to the rostral-most claustrum section, which was assigned a value of 0 mm. Note that not all subdivisions were present in every section examined. Scale bar = 0.5 mm in **(A–C,F–H)** and ~0.17 mm in **(D–E,I–J)**.

**Figure 5:**
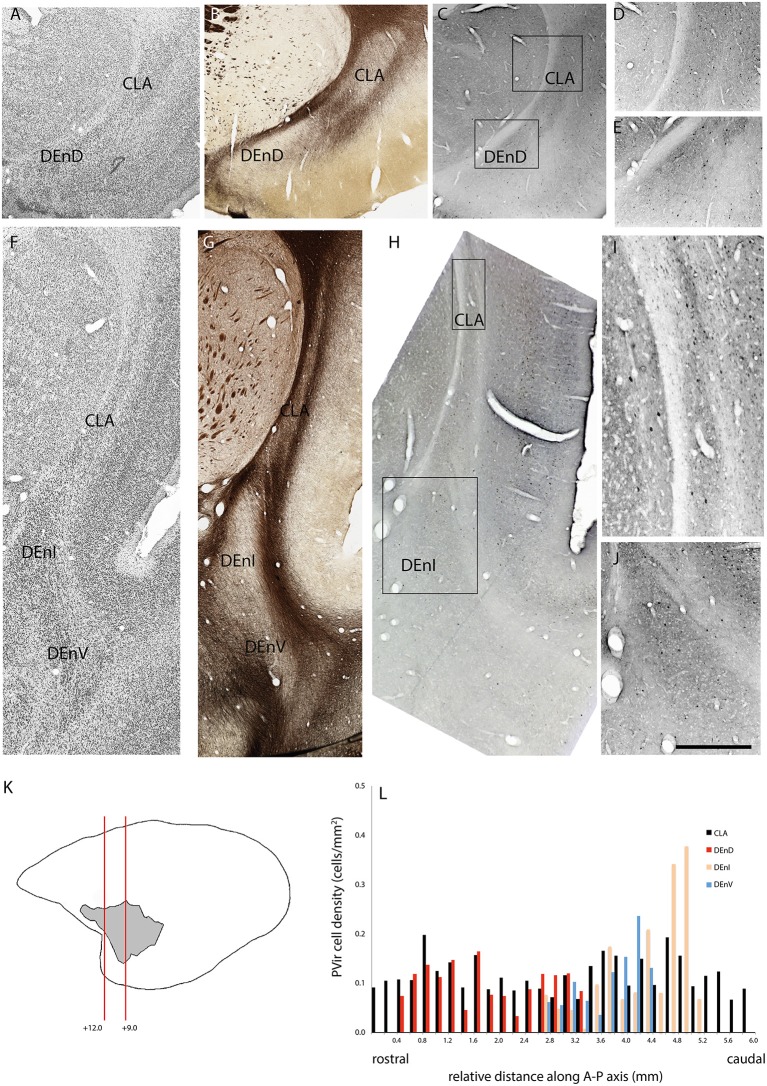
Adjacent rostral- **(A–E)** and mid- **(F–J)** coronal sections of the claustrum complex showing Nissl **(A,F)**; myelin **(B,G)**; and parvalbumin immunoreactive **(C–E,H–J)** staining in the CLA and DEnD subdivisions at each level from case F1882. **(K,L)** Figure annotations and conventions as in Figure 4.

In two cases, immunohistochemistry for calbindin (Figures 4A–L) or parvalbumin (Figures 5A–L) immunoreactive cell bodies was compared across the identified subdivisions of the claustrum complex. In each section containing CLA or endopiriform compartments, scanned images of the relevant sections were overlaid with the myelin-stained sections and internal boundaries identified. Calbindin (F1741) or parvalbumin (F1882) immunoreactive somae were counted, and the total was divided by the area of the respective compartment, as calculated in Adobe Illustrator (see “Materials and Methods” section). The resulting cell density was then plotted as a function of position along the anterior-posterior axis of the CLA, as shown in Figures 4E, 5E. Statistical comparison of immunoreactive cell densities was not possible, as only one case was examined for each protein, but some patterns were evident in the data. The density of both calbindin and parvalbumin-positive cells was highest in the DEnI compartments of caudal sections in both animals. Subjectively, there were also more calbindin immunopositive axons and terminals visible in the DEnI and DEnV compartments of case F1741, although this could not be easily quantified.

### Tractography of CLA and DEnD Compartments

**Figure 6:**
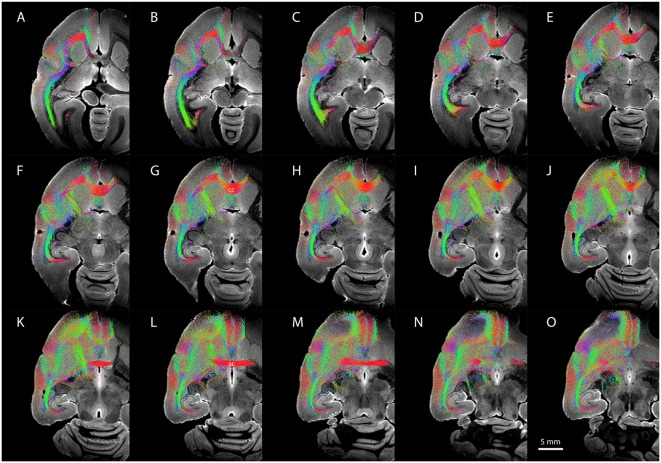
Diffusion-weighted imaging (DWI) tractography showing streamlines from the entire claustrum complex in case CJ197. (**A–O)** Horizontal sections showing fiber orientation distribution (FOD) for left cerebral hemisphere. Note high streamline density in orbital prefrontal cortex **(A–F)**, insular cortex **(D–H)**, and auditory cortex **(F–K)** lateral to the corpus callosum (labeled cc **in G**), and in medial prefrontal cortex **(K–O)** rostral to the anterior commissure (labeled ac **in L**).

Figure 6 shows representative DWI sections with claustrum complex-derived (all subdivisions) streamlines color-coded by anatomical orientation (red: medial-lateral; green: anterior-posterior; blue: dorsal-ventral) from the left hemisphere of case CJ197. High streamline density is evident in the medial-lateral direction in the genu of the corpus callosum (Figures 6C–J) and in the anterior commissure (Figures 6K–N) and in the anterior-posterior direction in the optic radiations and middle temporal lobe areas (Figure 6 all panels, especially panels  6A–C).

**Figure 7:**
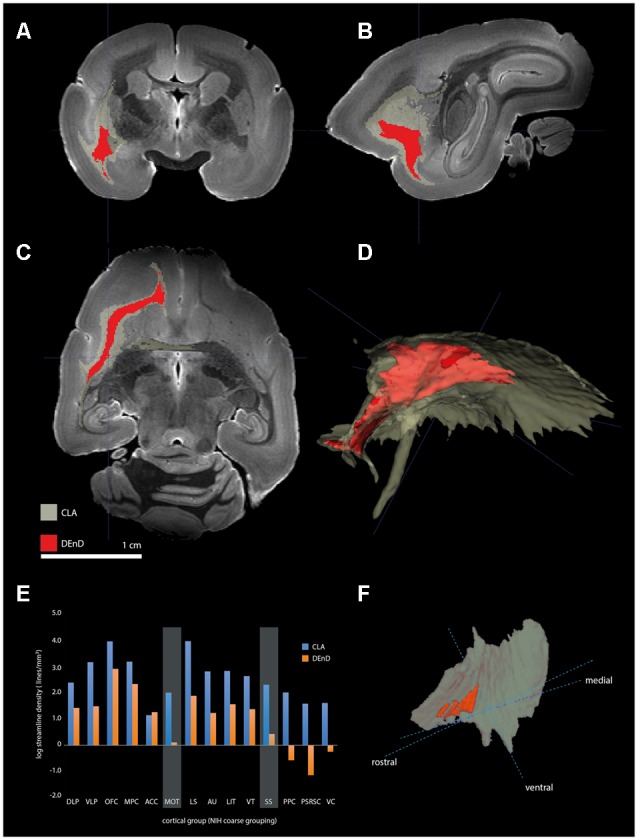
Tractography of claustrum complex vs. DEnD ROIs. **(A–C)** Coronal **(A)**, sagittal **(B)**, and horizontal views of streamline patterns emerging from the claustrum complex and dorsal division of the DEnD masks generated from the myeloarchitecture of case CJ197. Blue cross indicates a common point of registration in **(A–C)**. **(D)** Three-dimensional reconstruction of the streamline pattern in the left hemisphere white matter. The relative size of the seed ROIS is shown in the claustrum complex reconstruction in **(F)**. **(E)** Bar chart shows the log streamline density to each cortical region outlined in the coarse parcellation of the NIH marmoset atlas (see text for details). Disparity in the relative density of streamlines between the DEnD and remainder of the claustrum complex are highlighted for the motor (MOT) and somatosensory (SS) aggregated areas. The reduction in streamline density between DEnD and caudal cortical aggregates is evident in the posterior parietal (PPC), posterior cingulate/retrosplenial (PSRC), and visual cortex (VC) aggregates, where DEnD connectivity was negligible or absent in this single case.

Using the masks for the CLA and DEnD segmentations as generated from the myelin-stained sections for case CJ197, we compared the streamline density to all cortical regions by DWI (see “Materials and Methods” section). Relative streamline density from seed regions generated using 3-dimensional CLA and DEnD models is shown in Figures 7A–D. Reconstructions of the CLA (olive) and DEnD (red) models are shown in Figure 7F. Cortical regions were aggregated according to the 13-division coarse structure used in the NIH marmoset cortex atlas (Liu et al., 2018), with one modification. The anterior cingulate region (ACC), consisting mainly of area 24 (inclusive of all subdivisions) was separated from the medial prefrontal cortex region (MPFC), yielding a total of 14 cortical aggregate regions. The rationale for this adjustment was the differential claustrum connectivity between the MPC and ACC (Reser et al., 2017). Figure 7E shows the logarithmic streamline density for CLA and DEnD, as adjusted for the difference in volume between these subdivisions of the claustrum complex. A clear pattern which emerged from this analysis was a rostral-caudal gradient in streamline density from the DEnD subdivision, such that the caudal areas including posterior parietal (PPC), posterior cingulate/retrosplenial (PSRSC), and visual (VC) cortices exhibited almost no DWI connectivity with DEnD (<0.001 streamlines/mm^3^), despite moderately dense connectivity with the CLA mask. Motor and somatosensory cortex (MOT and SS, respectively) also exhibited a disparity between DEnD and CLA, with proportionally greater connectivity to the CLA subdivision. In contrast, the aggregated regions exhibiting the greatest streamline density from DEnD included prefrontal regions, especially orbitofrontal (OFC) and medial prefrontal (MPC) cortex, which were also among the regions most densely connected with CLA. Although these connectivity patterns were robust, and were internally consistent even when analyzed as 107 separately defined cortical areas according to the NIH Connectivity Atlas tool, they should be viewed as preliminary, as this analysis could only be performed on a single case (CJ197; see “Materials and Methods” section).

## Discussion

The claustrum has emerged as a focus of intense research interest (Baizer and Reser, 2017), for both its putative role in normal brain functions (Atlan et al., 2018; Bray, 2018; Jackson et al., 2018; White and Mathur, 2018a,b) and for its association with a number of devastating neurological and psychiatric diseases (Smythies et al., 2014; Patru and Reser, 2015; Marek, 2018), including epilepsy (Bayat et al., 2018; Silva et al., 2018; Zhang et al., 2018); frontotemporal dementia and amyotrophic lateral sclerosis (De Reuck et al., 2014, 2017); major depressive disorder (Su et al., 2018); schizophrenia (Cascella et al., 2011; Mallikarjun et al., 2018), Wilson’s disease (King et al., 1996), and Parkinson’s disease (Sener, 1998; Braak et al., 2001, 2007; Kalaitzakis et al., 2009; Shao et al., 2015; Sitte et al., 2017; Arrigo et al., 2018).

The molecular basis of claustrum function is starting to become clear (Kivell et al., 2014; Tantra et al., 2018), and both structural and functional connectomic studies have highlighted the importance of the claustrum complex for understanding connectivity in both normal and pathological brain states (Fernández-Miranda et al., 2008; Reser et al., 2014, 2017; Bruni et al., 2018; Fillinger et al., 2018; Rabellino et al., 2018; Randerath et al., 2018; Ribas et al., 2018; Stepniewska et al., 2018). However, there have only been a few coarse attempts to describe the extrinsic connectivity of the claustrum in terms of its internal structure (LeVay and Sherk, 1981; Pearson et al., 1982; Macchi et al., 1983; Tanné-Gariépy et al., 2002; Gattass et al., 2014; Reser et al., 2014), and the phylogenetic relationships of various components of the claustrum complex have only recently been described (Smith et al., 2019). In general, connectional studies have described both a rostral-caudal and dorsal-ventral topography of claustrum connections with respect to (primarily) cortical connections, but have not identified differential connectivity of e.g., the claustrum vs. endopiriform components of the claustrum complex. Here, we describe in detail the parcellation and nomenclature of the component nuclei of the marmoset claustrum complex, in order to better understand how the internal structure of the claustrum may inform connectional and functional anatomical studies.

Two key findings emerge from this work: (1) our data support an organization of the marmoset claustrum into two broad nuclei—the insular claustrum and the DEnD, with the latter being further segregated into three subdivisions, the dorsal, intermediate, and ventral endopiriform divisions; and (2) cortical areas exhibit differential connectivity, as indicated by DWI streamline density, between the CLA and the DEnD. Rostral cortical areas, especially the orbital and medial prefrontal cortex, exhibit strong connections to both the CLA and DEnD, while motor and somatosensory cortical areas are moderately to densely connected with the CLA, but exhibit less dense connectivity with DEnD. Finally, the cortical connectivity of DEnD shows a pronounced rostral-caudal gradient, such that the caudal VC, posterior cingulate, and retrosplenial areas have negligible DEnD derived-streamline connections, despite moderate to strong connectivity with the CLA. The latter observation, although it was clear from the DWI data, and resulted from long duration, high field strength scanning of the post-mortem tissue, should be regarded as preliminary, as it results from scanning of a single case. Furthermore, it was not possible in this instance to perform the same streamline analysis on the other subdivisions of the endopiriform nucleus, due to residual tissue damage to the posterior cortical regions arising from *in vivo* electrophysiology recordings.

Recent work has also described the chemoarchitecture of the marmoset claustrum and the distribution of a number of genetic and molecular markers with respect to the proposed structure of the claustrum complex (Watakabe, 2017). Characterization of the developmental origins and embryonic gene expression patterns of the claustrum complex has also been described recently (Watson and Puelles, 2017; Binks et al., 2019). Our findings regarding the internal subdivisions of the claustrum complex are largely in accordance with the insights of Watakabe et al. (2014), who showed a gradient distribution of neurogenetic markers along the principal axes of the marmoset claustrum. They suggested that although there are homologs of both the dorsal “insular” claustrum and the DEnD in the non-human primate brain, there may be functional overlap between these structures. Thus, the concept of a claustrum complex, as opposed to separate claustrum and endopiriform nuclei, represents the best available model of this structure across the mammalian order.

Interestingly, neither we nor Watakabe were able to identify a clear marmoset homolog of the rodent ventral endopiriform nucleus. This has led to the suggestion that there may be a closer relationship between the claustrum and amygdaloid complexes than previously thought, with the ventral endopiriform nucleus partially or completely subsumed within one of the peripheral amygdaloid structures (Smith et al., 2019). Confirmation of this speculation will require identification of one or more claustrum/endopiriform specific neuromarkers. Binks et al. (2019) and Watson and Puelles (2017) have also substantiated the division of the primate claustrum complex into two main compartments (insular claustrum and the DEnD), and have further clarified that both arise from the lateral pallium during development, albeit with different time courses and cellular migration patterns. Furthermore, they reported in both studies that the developmental origin of the as yet unidentified primate homolog of the rodent ventral endopiriform nucleus is the ventral pallium, reinforcing its exclusion from the organizational structure proposed for the claustrum complex.

Within the identified CLA in marmosets, we recognize four distinct subcompartments—a claustrum compartment, and three subdivisions of the DEnD. The most prominent distinguishing features of these compartments in our sample were differential myelin density and the orientation of myelinated axons. As most previous studies have relied exclusively on cytoarchitectonic, chemoarchitectonic, or connectional parcellation of the primate claustrum, the morphological identification of compartmental boundaries we describe may have been complicated by the absence of myeloarchitectonic data.

## Conclusion and Future Directions

This article highlights the high degree of uncertainty which persists with respect to the structure and function of the mammalian claustrum complex, especially among species in the primate order. While past studies have emphasized the apparent homogeneity of this forebrain nucleus, we show here that there is clear internal compartmentalization of the structure in a species with well-established homology of brain structure to Old World monkeys and humans. Understanding the regionalization of claustrum complex organization is important for gaining a clearer understanding of neurological diseases associated with disruptions of claustrum function. In addition, our work highlights the potential for complementary assessment of classical histological methods and modern, high field strength imaging techniques. Future directions for research will include deep investigation of connectivity between claustrum complex subdivisions and cortical areas, as well as other subcortical nuclei. Ideally, this will extend to studies of functional connectivity and network function *in vivo*.

## Data Availability Statement

The datasets generated for this study are available on request to the corresponding author.

## Ethics Statement

The materials used in the present study were obtained in accordance with the Australian Code for the Care and Use of Animals for Scientific Purposes (“the Australian code”), which explicitly encourages use of post-mortem tissue scavenged from animals used for approved research purposes. Eight post-mortem marmoset brains were obtained from adult marmosets involved in other experiments in our laboratory (Atapour et al., 2017, 2018), for which approval was obtained from the Monash University Animal Research Platform (approved protocol# MARP_2017-018).

## Author Contributions

DR provided funding and supervision for the project. DR and XP conceived the project, collected and analyzed study data, and wrote the manuscript. DW performed and analyzed MRI scans. NA performed and analyzed Neu-N stains. KJW and KHW performed histological procedures, assisted with surgical preparation and maintained animal records. JC assisted with histological procedures and photography of specimens. AR assisted with histological preparations and analysis. MR provided expert anatomical assistance, data quality checks, and assisted with editing of the final manuscript.

## Conflict of Interest

The authors declare that the research was conducted in the absence of any commercial or financial relationships that could be construed as a potential conflict of interest.

The reviewer RO declared a past co-authorship with one of the authors DR to the handling Editor.

## Footnotes

^1^www.mrtrix.org

^2^http://www.itksnap.org

^3^http://stnava.github.io/ANTs/

^4^http://blog.k8baldwin.com/Finding-areas-and-lengths-in-Illustrator-td4.html
